# Alternative routes to HiAP implementation

**DOI:** 10.1093/eurpub/ckac129.577

**Published:** 2022-10-25

**Authors:** M Bekker

**Affiliations:** 1 EUPHA-PHPP; 2 Center for Space, Place and Society, Wageningen University, Wageningen, Netherlands

## Abstract

From a public health political science perspective there are alternative routes to HiAP. Besides the ‘classic’ HiAP approach seeking government internal departmental alignments, facilitating and rewarding bottom up social initiatives by communities, or marketed health innovations by commercial health-related consultancies and enterprises, and regional (socio-) economic innovation networks that after a developmental stage can start to pressure multi-level governments for action. A final route lies along the litigation path (Think of climate agreements and governments being held accountable by the judiciary to comply with agreed emissions reductions etc.)

